# Cutaneous mucormycosis postcosmetic surgery: A case report and review of the literature

**DOI:** 10.1097/MD.0000000000004185

**Published:** 2016-07-08

**Authors:** Khaled Al-Tarrah, Mahmoud Abdelaty, Ahmad Behbahani, Eman Mokaddas, Helmy Soliman, Ahdi Albader

**Affiliations:** a Department of Burns and Plastic Surgery; b Department of Microbiology, Ibn Sina Specialist Hospital; c Department of Microbiology, University of Kuwait, Kuwait.

**Keywords:** cosmetic surgery, cutaneous mucormycosis, fat grafting, iatrogenic, lipofilling

## Abstract

**Background:**

Mucormycosis is a rare, aggressive, and life-threatening infection that is caused by organisms belonging to the order Mucorales. It is usually acquired through direct means and virtually always affects immunocompromised patients with the port of entry reflecting the site of infection, in this case, cutaneous. Unlike other mucormycoses, patients affected by *Apophysomyces elegans (A elegans)* are known to be immunocompetent. This locally aggressive disease penetrates through different tissue plains invading adjacent muscles, fascia, and even bone causing extensive morbidity and may prove fatal if treated inadequately. Cutaneous mucormycosis is associated with disruption of cutaneous barriers such as trauma. However, rarely, it may be iatrogenic. No cases have been previously reported postcosmetic surgery, especially one that is so commonly performed, lipofilling.

**Case Report:**

The patient is a, previously healthy, 41-year-old middle-eastern female who was admitted to the plastic surgery department 17 days after undergoing cosmetic surgery. She suffered from extensive tissue inflammation and necrosis in both gluteal regions. Following admission, she was initially started on empirical antimicrobial therapy which was changed to an antifungal agent, voriconazole, when preliminary microbiological results showed filamentous fungi. This was discontinued and liposomal amphotericin B was commenced when further mycological analysis identified *A elegans*. Furthermore, she underwent a total of 10 sessions of extensive debridement to the extent that portions of the sacrum and left femoral head became exposed. Her clinical status and wounds improved with the appropriate management and she remained an inpatient for 62 days. Subsequently, she had defects in both gluteal regions which required reconstructive surgery.

**Conclusion:**

*A elegans* is an uncommon cause of iatrogenic cutaneous mucormycosis. A high index of clinical suspicion is required, especially in the absence of clinical improvement despite conventional methods of treatment, so that early diagnosis can be reached and the appropriate management instigated promptly in order to mitigate morbidity and mortality. Reversal of predisposing risk factors, regular extensive surgical debridement, and antifungal therapy remain the cornerstones of therapy for this life-threatening condition.

## 1 Introduction

*Apophysomyces elegans (A elegans)* is a saprophytic fungi belonging to the order Mucorales. It is omnipresent in the environment such as soil and decaying vegetation and is known to cause mucormycosis. Mucormycosis is a rare, aggressive, and life-threatening infection that usually occurs when spores from the molds are acquired directly via ingestion, inhalation, or inoculation (usually traumatic), and almost always affects the immunocompromised. The port of entry reflects the site of infection with the most common being sinus disease (39%), followed by pulmonary (24%), cutaneous (19%), cerebral (9%), other sites (9%), and gastrointestinal (7%), respectively, and is associated with an overall mortality ranging from 17% to 66% depending on predisposing risk factors.^[1]^ Unlike other mucormycosis, the majority of patients infected with *Apophysomyces* species are immunocompetent.^[2]^

The prerequisite for acquiring cutaneous mucormycosis appears to be the disruption of the cutaneous barriers, either through traumatic or iatrogenic means. Dressings, intravenous cannulation, injections, and surgeries have all been implicated.^[3]^ Surgical site infections accounted for 41% of healthcare-associated mucormycosis, commonly associated with cardiovascular surgery, followed by gastrointestinal, ophthalmic, urological, and orthopedic surgery.^[4]^ Cutaneous mucormycosis can be locally aggressive penetrating the cutaneous and subcutaneous plains and invading adjacent muscle, fascia, and even bone causing extensive morbidity and may even prove fatal if treated inadequately.

To our knowledge, no cases have been reported where mucormycosis has developed as a result of undergoing cosmetic surgery especially one that is so commonly performed, lipofilling.

## 2 Case report

The patient is a 41 year old middle-eastern female who underwent abdominoplasty, liposuction (arms and back), and buttock augmentation by fat grafting in the private setting. Her inpatient stay was uneventful and was discharged home 2 days postoperatively. She returned for follow-up on day 4 postoperatively, with concerns regarding a red, hot, and tender swelling in the left gluteal region. She was then admitted to the hospital for 2 days where intravenous ceftriaxone was started. She underwent surgical debridement of necrotic subcutaneous tissue the next day. She continued to attend her outpatient clinic appointments for a few days but was readmitted with similar complaints. Intravenous ceftriaxone was administered and she underwent further debridement of necrotic tissues. Postoperatively, intramuscular chymotrypsin and daily dressings were commenced. Clinically, her condition continued to deteriorate and she became febrile with peripheral temperature readings of 38 °C and started to develop gangrenous wounds. On day 6 postadmission, the patient was then transferred to the operating theater where further necrotic tissue was debrided. On day 17 postcosmetic surgery, she was transferred to the plastic surgery department with a specialist public hospital.

The patient was previously healthy with unremarkable past medical, surgical, and family histories. Her concomitant medications were related to her initial surgery and included analgesics, antibiotics, and omeprazole. She has no known drug allergies. She denies alcohol intake, smoking, and recreational drug use. She only traveled to Austria, Germany, and Netherlands 3 months prior to undergoing cosmetic surgery. She denies other risk factors such as being exposed to any environmental sources of infection such reconstruction/demolition sites, animal/insect bites, or any form of penetrating injury.

**Figure 1:**
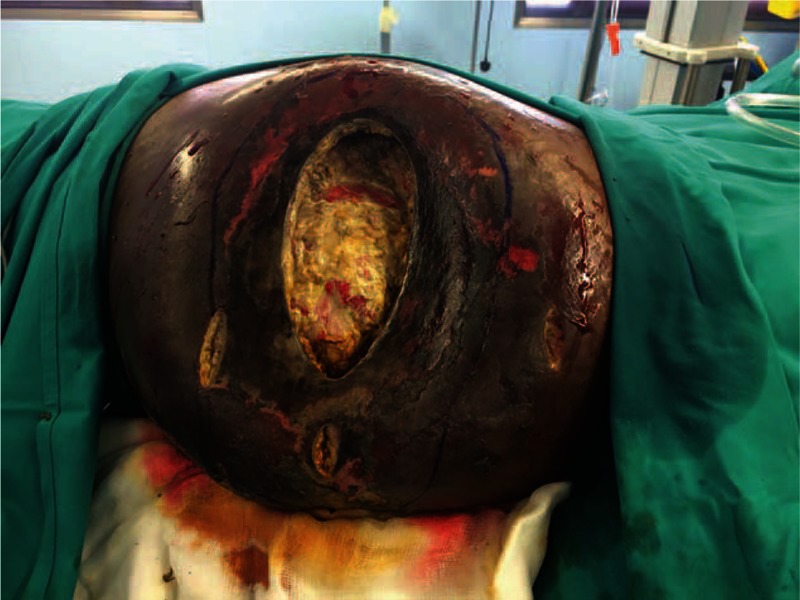
Wounds prior to the 1st surgical debridement in Ibn Sina Specialist Hospital.

On admission, the patient's vital signs and systemic examination were unremarkable. The gluteal regions were severely affected, there was a 20 cm by 15 cm wound at the left buttock with extensive necrotic skin and subcutaneous tissue. The right buttock was indurated and swollen and other operative sites were unremarkable. Laboratory investigations showed hemoglobin levels of 8.1 (g/dL), white cell count of 46. 69 (10^9^/L), and procalcitonin levels of 1. 46 (ng/mL), other tests were unremarkable (Fig. 1).

**Figure 2:**
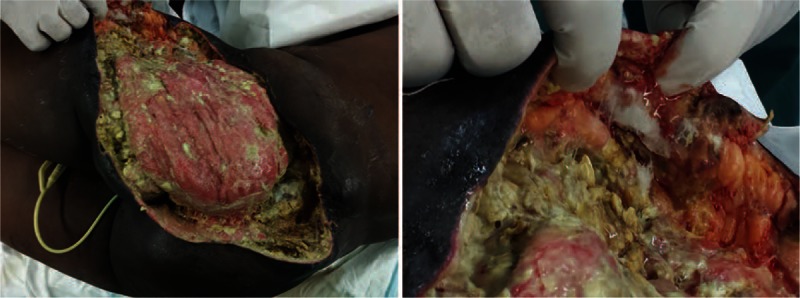
Appearance of the wound during the 4th surgical debridement. Colonies of the organism present within different regions of the wound.

The patient's management involved regular analgesics, empirical intravenous Meropenem, and surgical debridement of her wounds. Despite this, she continued to be febrile with temperatures of >39 °C and her inflammatory markers (including procalcitonin levels) continued to rise. Upon dressing the following day, new areas of necrosis have developed. She continued to undergo regular extensive surgical debridement, almost twice weekly, to the extent that portions of the sacrum and head of femur became exposed. Vacuum-assisted closure therapy (V.A.C ATS^®^/GranuFoam Silver^®^, KCI San Antonio, TX) was applied and adjusted to 100 mm Hg continuous mode following debridement. Regular tissue samples were sent to histopathological and microbiological analysis. At 2 weeks postadmission, preliminary microbiological results showed filamentous fungus, prompting the initiation of intravenous voriconazole empirically at 450 mg (6 mg/kg) twice daily for 24 hours followed by 300 mg (4 mg/kg) every 12 hours thereafter. This was continued for 2 weeks and was changed accordingly upon receiving the full mycology report stating the presence of *A elegans*. This was also confirmed by histopathology samples. Liposomal amphotericin B (AmB) was then started accordingly at a dose of 375 mg (5 mg/kg) per day for a further 3 weeks (Fig. 2).

**Figure 3:**
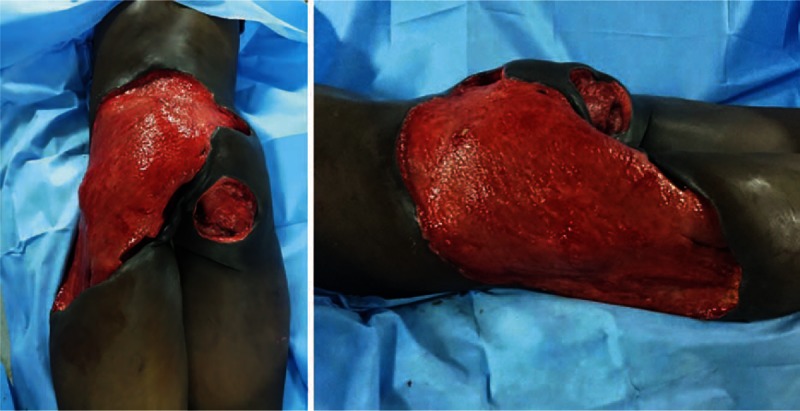
Final appearance of the wounds after multiple debridements.

After commencing the appropriate antifungal therapy and several debridement procedures, the patient's general condition, vital signs, investigations, and wounds have gradually improved. She underwent a total of 10 surgical interventions. Her final wounds had healthy granulation tissue with no signs of infection and were deemed suitable for surgical reconstruction (Fig. 3).

## 3 Discussion

*A elegans* was first isolated from the soils of India in 1979.^[5]^ It is distinguished from other Mucorales by having funnel or bell-shaped apophyses, thin sporangiosphores with hemispherical columellae, and foot cells. It is also recognized for its thermotolerance, rapidly growing in temperatures reaching up to 42 °C.^[6]^ Over the last 2 decades, there has been an increasing trend of *A elegans* being isolated from patients living in tropical and subtropical climates with the majority of reported cases, approximately 60%, being from India and the rest from the United States, Australia, Mexico, Caribbean Islands, Columbia, and Venezuela.^[2]^ It has also been implicated as a cause of mucormycosis following natural disasters within the vicinity of the above regions, such as a tsunami.^[7]^

Majority of patients affected by *A elegans* tend to develop cutaneous and soft-tissue infections.^[8]^ Cutaneous mucormycosis typically requires the skin to be damaged and hence, its function as a barrier disrupted. This is usually achieved through traumatic means followed by subsequent contamination from environmental sources such as soil and water. On rarer occasions, the cause of the disease and transmission of the organism has been iatrogenic. Nosocomial mucormycosis has been associated with a variety of procedures including dressings and injections.^[9]^ Most likely the procedures were undertaken using contaminated medical equipment or dressings resulting in the implantation of the pathogen.

Roden et al^[1]^ reported that surgery accounted for 15% of 929 mucormycosis cases published. Two cases where *A elegans* was identified as the culprit organism were described in the literature. In 1992, a 27-year-old male developed “postoperative necrotizing fasciitis” after undergoing inguinal herniorrhaphy. Despite extensive surgical and medical management, the patient died.^[10]^ Another case was reported in 1995, a 32-year-old female was admitted to hospital 10 days after undergoing caesarian section with intermittent fever, pain, swelling, and discharge near the surgical scar. Aggressive management was started in the form of empirical antibacterial therapy and surgical debridement. Once laboratory investigations were consistent with mucormycosis, AmB was started. The patient survived and was discharged 2 months later.^[11]^

Due to the thermophilic properties of *A elegans*, once inoculated, it aggressively invades different tissue plains resulting in dissemination or the formation of chronic infection. Histopathological findings are similar to that of other mucormycoses. The morphological features of the Mucorales is present within the affected tissues and is associated with a mixed suppurative and necrotizing inflammatory reactions. The hyphae or hyphal fragments, with its distinctive right angle branching, gradually invade adjacent tissues including the walls and lumens of blood vessels. Due to this angioinvasion, thrombosis subsequently develops resulting in hemorrhagic infarction of tissues. This most likely clarifies the rapid progression of tissue necrosis, which usually occurs within days of inoculation and hence the extensive morbidity and mortality rates. Therefore, it is imperative to treat mucormycosis aggressively in order to improve prognosis. There are 4 critical factors to consider: rapidity of diagnosis, reversal of underlying risk factors if possible, aggressive surgical debridement, and appropriate antifungal therapy.^[12]^

The diagnosis of mucormycosis requires a high index of clinical suspicion and relies on identification of risk factors, clinical findings as well as histopathological and microbiological analyses. Several risk factors are associated with cutaneous mucormycosis, including breach of skin barrier, diabetes, and immunosuppressive states (e.g., malignancy, iatrogenic, etc…).^[1,13]^ Clinical manifestations include pain, erythema, and tissue swelling progressing into abscess formation and eventually the development of tissue necrosis within the infected area. The overlying skin usually appears red and indurated and may ultimately become gangrenous, forming black eschars. As in this case, fungal colonies may be present within the wound. Taking into account the dramatic clinical course of the disease, the possibility of mucormycosis must be considered as a differential diagnosis. Especially, when the response to antibacterial therapy is absent so that the necessary investigations and aggressive management can be initiated promptly.

Unfortunately, as there are currently no quick reliable diagnostic investigations for mucormycosis, direct examination and culture-based methods remain crucial. Therefore, lesion biopsies are essential in order to establish a definitive diagnosis. Tissue samples should be taken from the center of the lesion and include subcutaneous fat, as the mold frequently invades the vasculature of the dermis and subcutis, resulting in an ischemic cone at the skin surface.^[14]^ The colonies of Mucorales, in this case *A elegans*, are usually cultured using Sabouraud dextrose agar and incubated in temperature ranging between 25 and 42 °C and are typically described rapidly growing, creamy white and fluffy in appearance with abundant aerial mycelia.^[6,10]^ Unfortunately, these investigations take time to be fully processed which may prove fatal.

Up to half of mucormycosis cases, in general, are diagnosed postmortem.^[15,16]^ Cutaneous disease has an overall mortality rate of 31% and when associated with dissemination, the mortality rate can reach up to 94%.^[1]^ This highlights the critical need to maintain a high index of suspicion, so that rapid aggressive management can be initiated promptly. Early diagnosis is also essential because small focal lesions can often be excised surgically before the disease invades critical structures or disseminate.^[17]^

Management of mucormycosis includes systemic antifungal therapy in conjunction with radical extensive surgical debridement. AmB deoxycholate and isavuconazonium sulfate are the only drugs licensed by the Food and Drug Administration for the treatment of mucormycosis. However, AmB deoxycholate use is limited due to its nephrotoxicity and infusion-related side effects. This has been supplanted by lipid formulations of AmB which are significantly less nephrotoxic and can be safely administered at higher doses over longer periods of time. It has also been associated with improved survival rates (67%) when compared to AmB deoxycholate (39%).^[18]^ Although optimal dosing regimen remains unclear, 3 to 5 mg/kg/day should be used.

Posaconazole, a triazole, can be used as secondary prophylaxis following successful management with AmB or as active treatment for cases in which AmB therapy failed or was not tolerated.^[19–21]^ Prophylactic treatment involves oral administration of 300 mg twice daily for the 1st 24 hours, followed by 300 mg once a day thereafter. The intravenous form is used for active management, utilizing the same dosing regimen. In cases where both AmB and posaconazole therapy has been unsuccessful or not tolerated, isavuconazonium is an alternative treatment option.^[21–23]^

Antifungal therapy alone is usually inadequate to control mucormycosis. The hallmark angioinvasion, ischemia, and tissue necrosis may result in poor penetration of the antifungal agent to the infection site. Therefore, even if the pathogen is susceptible to the antifungal agent in vitro, it may be ineffective in vivo. Furthermore, killing the organism may not prevent the massive amount of tissue necrosis that occurs during the course of the disease.^[24]^ Therefore, prompt extensive surgical debridement is necessary. The amount of debridement procedures required may be numerous, with an average of 10 sessions usually performed.^[25]^ Occasionally, amputations may be necessary to save the patient's life.^[26]^ Due to the nature and extent of the surgical debridement required, if the patient survives the acute stage, major reconstructive surgery may be necessary to manage the resulting highly disfiguring defects.

Mortality rates of mucormycosis are dismal if left untreated, with survival rates of 3% when compared to cases where treatment was initiated in the form of antifungal therapy alone, surgical therapy alone, or combined therapy (62%, 57%, and 70%, respectively).^[1]^

## 4 Conclusion

*A elegans* is an uncommon cause of iatrogenic cutaneous mucormycosis. A high index of clinical suspicion is required, especially in the absence of clinical improvement despite conventional methods of treatment, so that an early diagnosis can be reached and the appropriate management instigated promptly in order to mitigate morbidity and mortality. Reversal of predisposing risk factors, regular extensive surgical debridement, and antifungal therapy remain the cornerstones of therapy for this life-threatening condition.

## 5 Consent

Written informed consent was obtained from the patient for publication of this case report and any accompanying images. A copy of the written consent is available for review.

## Acknowledgments

The authors thank Dr Qutaibah Alkandari and Prof Ziauddin Khan for their input and assistance provided during the treatment of the patient, as well as their support in writing the manuscript.
